# Parenting After Child Maltreatment: A Study of Maternal Parenting Strategies and the Parent-child Relationship

**DOI:** 10.1007/s40653-026-00850-1

**Published:** 2026-03-16

**Authors:** Marit Burkeland-Lie, Jens Christoffer Skogen, Børge Sivertsen, Kaia Kjørstad, Anders Dovran, Gertrud Sofie Hafstad, Mari Hysing

**Affiliations:** 1 Department of Health Promotion, Norwegian Institute of Public Health, Bergen, Norway; 2 Department of Psychosocial Science, Faculty of Psychology, University of Bergen, Bergen, Norway; 3 Center for Alcohol & Drug Research, Stavanger University Hospital, Stavanger, Norway; 4 Department of Research & Innovation, Helse Fonna Hospital Trust, Haugesund, Norway; 5 Stine Sofies Foundation, Grimstad, Norway; 6 Norwegian Centre for Violence and Traumatic Stress Studies, Oslo, Norway

**Keywords:** Parent-child relationship, Parenting strategies, Violence, Abuse, Childhood maltreatment

## Abstract

Parenting practices and the parent-child relationship play a crucial role in child development. Families affected by child maltreatment may experience poor parent-child relationships and negative parenting practices. Research on parenting where both mothers and children have experienced maltreatment is limited. This study aims to investigate the parent-child relationship and parenting strategies among mothers (*N* = 228) of children (5–11 years) with maltreatment experiences, and whether there are differences in parenting behaviors between mothers with and without personal maltreatment histories. The Alabama Parenting Questionnaire (APQ) was used to assess parenting strategies, while the Child-Parent Relationship Scale (CPRS) was utilized to investigate the parent-child relationship. Mean scores and standard deviations were calculated for the entire sample and the two groups of mothers. To compare the groups, we used two-sample t-tests and generated plots to illustrate response distributions. Fisher’s exact test was also used to compare response patterns. We found no significant differences between the groups in the APQ subscales (positive parenting and inconsistent discipline) or the closeness subscale of the CPRS. Both groups reported using positive parenting strategies and having close relationships with their children. However, mothers with maltreatment experiences reported significantly higher conflict (CPRS) scores than those without such experiences (*p* = 0.009). Clinicians can build on the strengths observed in this study to enhance the parent-child relationship and parenting practices. The elevated conflict levels reported by mothers with maltreatment experiences highlight a critical area for interventions aimed at supporting healthier family dynamics and reducing conflict.

## Introduction

*Child maltreatment* is defined by the World Health Organization (2024) as the abuse and neglect of children under the age of 18 years, covering all types of physical, emotional, and sexual abuse, neglect, and exploitation. It is a significant public health problem and a key contributor to health inequalities (Sethi et al., 2013). However, research in the area is often demanding and complex due to ethical and practical challenges, including societal taboos and legal constraints regarding parental consent (Jackson, 2023; Chae et al., 2011; Kinard, 2001). These challenges often hinder the recruitment of children exposed to maltreatment and their families.

Attachment theory suggests that a stable and secure relationship with a caregiver beginning in infancy supports a strong sense of self and fosters positive developmental outcomes (Thompson, 2024). In the context of child maltreatment, this type of parent-child relationship may serve as a protective factor, mitigating the impact of trauma on behavioral and emotional development (Harden et al., 2024). Studies on the moderating role of the parent-child relationship quality have shown that it can play an essential role in influencing the impact of child maltreatment, with stronger parent-child relationships often reducing the severity of behavioral and emotional difficulties in children (Fagan, 2020; Steele & McKinney, 2020).

In a study of non-offending parents of children (ages 4–16 years) exposed to physical abuse, sexual abuse, or neglect, the parents of exposed children reported poorer parent-child relations, greater family disengagement, and more chaotic family functioning than parents of non-exposed children (Cabbigat & Kangas, 2018). To our knowledge, few studies have explored how adolescents with childhood maltreatment experiences perceive their parents’ behavior and family dynamics. For example, in a Swedish study including adolescents and young adults (ages 16–22 years; *n* = 3288), those who had experienced physical abuse perceived their parents as having a more controlling and less affectionate parenting style compared to peers with no abuse experience (Nilsson et al., 2017). Other studies have found that adolescents (ages 12–18 years) with a history of physical abuse reported dysfunctional family relationships and poorer family cohesion and rated their parents’ parenting style as more negative with lower levels of care compared to adolescents with no physical abuse experiences (Pelcovitz et al., 2000; Sunday et al., 2008).

Many factors influence parenting behaviors, including social and economic climates and parents’ cultural background, mental health, and social support (Roubinov & Boyce, 2017; Fung et al., 2018; Sanders & Turner, 2018). Child characteristics may also affect parenting approaches. For instance, in the context of families affected by child maltreatment, some studies have found indications that non-offending parents of sexually abused children may experience intense feelings of guilt (Banyard et al., 2001; Vilvens et al., 2021). A possible consequence of the guilt could be overly lenient parenting (Vaughan-Eden, 2014). Elevated psychological distress due to the abuse of their children may also lead to less positive, consistent, and involved parenting (Harden et al., 2024). However, the complexity of influences on parenting behaviors makes determining whether the parenting was compromised before or after the maltreatment occurred difficult.

There is increasing interest in how maternal experiences of childhood maltreatment could potentially affect parenting practices, and many reviews have been performed in the last decades (for example, Hughes & Cossar, 2015, and; Christie et al., 2017). A review including studies on intergenerational effects of maltreatment found that childhood maltreatment experiences may alter parents’ ability to avoid harmful parenting practices and utilize positive ones (Greene et al., 2020). The systematic review found that parents’ childhood maltreatment experiences were consistently associated with being less emotionally nurturing and responsive toward their younger children. The results for parents of school-aged children and adolescents regarding being nurturing and available were mixed. Overall, while most parents who were abused as children do not abuse their children, the risk of them utilizing abusive parenting behaviors is substantial, and their maltreatment histories may alter their ability to apply positive parenting practices such as providing nurturing responses, encouraging learning and supporting positive peer relationships (Greene et al., 2020).

While much attention has been given to the association between childhood maltreatment and abusive or neglectful parenting, there is far less focus on the association with negative parenting behaviors such as being hostile, rejecting or using inconsistent discipline techniques (Greene et al., 2020). Additionally, it is essential to acknowledge that it remains uncertain whether the compromised parenting strategies and parent-child relationship are consequences of maltreatment and/or if they existed before the maltreatment took place.

Taken together, there is limited knowledge about parenting strategies and the parent-child relationship of parents with young children who have experienced maltreatment, as well as how maternal exposure to violence and abuse may relate to these factors. This study aimed to explore (1) the characteristics of parenting strategies and the parent-child relationship among non-offending mothers of children who have been exposed to violence and abuse, and (2) whether differences in parenting behaviors exist between mothers with experiences of violence and abuse and those without such experiences.

## Methods

### The Triple-S Study

The Norwegian *Triple-S* study is a longitudinal cohort study with a multi-informant design, collecting data from children with maltreatment histories, and their families (Schønning et al., 2021). The study aims to produce new knowledge on predictors and short- and long-term consequences of childhood maltreatment to inform policymaking, program planning, and service delivery. The Triple-S abbreviation originates from the Norwegian name Stine Sofie Senteret (The Stine Sofie Center, SSC), which is run by the non-profit organization the Stine Sofie Foundation (SSF). The SSC is a learning and coping center for children and adolescents aged 18 years or younger who have experienced physical or emotional abuse, neglect and/or sexual abuse. This includes experiences such as being pushed violently, struck, kicked, verbally degraded, threatened with violence, confined, or having an adult touch their private parts or force them to show their private parts (Schønning et al., 2024). Children and adolescents participate voluntarily in a one-week course at the SSC and can apply to participate with their caregiver, through their primary physician, Child Welfare Services, or other officials. Those attending with a biological parent participate with a non-offending parent.

### Procedure and Recruitment

The Triple-S recruitment phase commenced in January 2021 (Schønning et al., 2021). Study participants are recruited from the SSC upon entry to the center and provided with written information about the project from personnel. They are asked to complete a questionnaire and provide self-reported information. Caregivers provide information for the youngest children (5–11 years). Some participants had already visited the SSC before the recruitment phase began. These participants were recruited via e-mail, telephone calls, or through the SSC website and social media platforms and completed the questionnaire at home.

When the children and caregivers arrive at the center, researchers and trained health personnel are available to address any further questions. The personnel at the center are also available when the participants fill out the questionnaire. Participants are informed that their questionnaire data is confidential and that their participation in the study does not affect their participation in the one-week course at the center.

To be included in the present study, participants had to have a child aged 5–11 years with a history of maltreatment substantiated by their primary physician, Child Welfare Services, or other officials, be able to complete the questionnaire in Norwegian, report being the biological parent of the child, and have the child living primarily with them. The final analytic sample comprised 228 mothers. Although fathers were represented in the overall cohort (approximately 10%), they constituted a small minority in this age-specific subsample and were therefore not included in the present analyses.

### Compliance with Ethical Standards

The study was approved by the Regional Committees for Medical and Health Research Ethics (REK, #95445). All participants provided informed electronic consent and were informed that they could withdraw from the study at any point. They were assured that participation in the study would not affect their participation in the one-week course or any future contact with the SSC. During the data collection at the center, personnel from the SSC were present, and they were also available by telephone for participants to answer the questionnaire at home. All collected data was handled in accordance with the General Data Protection Regulation (GDPR). The study was performed in accordance with the ethical standards laid down in the 1964 Declaration of Helsinki and its later amendments.

### Background Variables

The participating mothers provided demographic information, including their age and country of birth. Age was determined using the first six digits of their Norwegian national identity number, corresponding to their birth date. Socioeconomic factors, such as educational level, employment status, and household income, were also assessed. Participants were provided with multiple response options to capture diverse forms of employment (e.g., full-time, part-time) and non-employment statuses (e.g., disability pension, sick leave), as well as temporary statuses (e.g., currently studying, seeking/applying for work). They reported their highest level of completed education, with options ranging from basic schooling (primary and upper secondary) to higher education (college or university). Household income was reported by selecting from nine predefined categories, ranging from “Below 250,000 NOK” (approximately 21,500 euros) to “2,000,000 NOK or more” (approximately 171,000 euros). In addition, participants were asked to assess their family’s overall economic situation on a 5-point scale, where 1 was “very poor”, 3 was “average,” and 5 was “very good”. Finally, they were also asked to report on their partner’s employment status, if applicable, to determine whether the household had more than one income.

### Assessing the Mothers’ Experiences

To assess the mothers’ experiences with maltreatment, questions developed for the *Nord-Trøndelag Health Study* (HUNT) were used. The HUNT study is a large longitudinal population health study in Norway that gathers information on a wide range of health conditions and lifestyle factors, including psychological distress, traumatic events and family cohesion (Broekhof et al., 2022). Specifically, we included questions from the Young HUNT 3 study conducted between 2006 and 2008, which focuses on young adolescents (Holmen et al., 2014). Participants were asked if they had been exposed to (1) violence or being beaten up; (2) sexual harassment; (3) rape; and (4) sexual abuse other than rape. The mothers could respond “yes” or “no” to each question. If they answered “yes,” they were asked follow-up questions, including the age of first exposure to the specific form of maltreatment. Based on their responses, the mothers were divided into the *Maternal exposure* and *No maternal exposure* groups. The former group (*N* = 160) included mothers who answered “yes” to having experienced at least one of the maltreatment categories, while the No Maternal Exposure group (*N* = 68) comprised mothers without such experiences.

### The Alabama Parenting Questionnaire

The *Alabama Parenting Questionnaire* (APQ) is used to assess parental involvement with their child (Frick, 1991). A modified version of the APQ was used in the present study to assess *positive parenting* and *inconsistent discipline*. The modified version consists of eight items, four in each subscale. Items are rated on a 5-point scale ranging from “never” (1) to “always” (5). A high score on the positive parenting subscale reflects a parenting practice characterized by positive involvement with the child. Conversely, a high score in the inconsistent discipline subscale indicates low consistency in discipline techniques, such as failure to follow through with consequences for misbehavior (Shelton et al., 1996).

The Cronbach’s alpha coefficients for positive parenting and inconsistent discipline in the Norwegian version of the original 42-item version of the APQ are 0.71 and 0.63, respectively (Sollie et al., 2016). In this version, positive parenting and inconsistent discipline have six items each. In another Norwegian study using the positive parenting subscale with six items, the Cronbach’s alpha was 0.81 (Brekke et al., 2023). The item “You reward or give something extra to your child for obeying or behaving well” from the positive parenting subscale negatively impacted the reliability of the subscale in our modified version and was therefore removed.

### The Child-parent Relationship Scale

The *Child-Parent Relationship Scale* (CPRS) measures the quality of the parent-child relationship (Pianta, 1992). In this study, we used the short version of the instrument with 15 items divided into two subscales: *conflict* (8 items) and *closeness* (7 items). Items are rated on a 5-point scale ranging from “definitely does not apply” (1) to “definitely applies” (5). The conflict subscale measures the degree to which parents perceive their relationship with their child to be characterized by negativity. In contrast, the closeness subscale measures the extent to which the relationship is characterized by warmth, love, and open communication. These two subscales measure distinct domains of the parent-child relationship, with a weak correlation (*r* = 0.16) between them (Driscoll & Pianta, 2011). The Norwegian version of the CPRS has demonstrated satisfactory validity and reliability, with Cronbach’s alpha being 0.83 for conflict and 0.82 for closeness, as shown in the International Child Development Programme (ICDP) study (Brekke et al., 2023).

### Statistical Analysis

The data were analyzed using R version 4.3.2 (R Core Team, 2023). First, we conducted descriptive analyses to present the sample, reporting frequencies and percentages. We also calculated the mean and standard deviation for the age at which the mothers first experienced maltreatment. Next, we performed Pearson’s correlation analyses to examine the relationship between the positive parenting and inconsistent discipline subscales in the APQ and the closeness and conflict subscales in the CPRS.

To compare our modified version of the APQ to the original Norwegian version (Sollie et al., 2016), we standardized mean scores for each version based on the maximum possible score (percentage score). We calculated standardized scores and their confidence intervals to assess variability and consistency, computed Cohen’s d to estimate effect sizes, and applied a significance test (p-value) to determine whether there were significant differences between the scores from the two versions.

We also calculated the mean and standard deviation for each subscale in the APQ and CPRS for the entire sample, as well as for the two groups of mothers (Maternal exposure and No maternal exposure groups). A two-sample t-test was performed to assess whether there was a statistically significant difference in mean scores between mothers with and without maltreatment experiences. Additionally, we calculated Cohen’s d for each subscale using the ‘effectsize’ package (Ben-Shachar et al., 2020).

To complement the subscale t-tests, we created plots illustrating the distribution of responses to each item in the APQ and CPRS subscales for the two groups of mothers. This method visualizes response patterns across multiple Likert-scale items, providing insight into how participants rated various statements. By visually comparing the response distributions between mothers with and without maltreatment experiences, we aimed to identify trends in the two groups and understand how exposure might influence parenting practices and the parent-child relationship. We also performed Fisher’s exact test to compare the two groups of mothers. The tables were produced using ‘gtsummary’ (Sjoberg et al., 2021), and the plots were generated using the ‘Likert’ package (Bryer & Speerschneider, 2016) in R.

## Results

### Sociodemographic Characteristics

This study included 228 mothers with a mean age of 39 years, ranging from 23 to 57 years. Their children had a mean age of 9 years, consisting of 55% girls and 45% boys. As detailed in Table 1, the majority (77%) of the participants were born in Norway, and education levels varied, with 33% having completed upper secondary education and 37% holding higher educational degrees. Regarding employment, 26% worked full-time, 16% worked part-time, and 37% were on sick leave, disability benefit, or work assessment allowance. Most participants reported a household income between 250,000 and 499,000 Norwegian kroner (roughly 21,500 − 43,000 euros), with 35% rating their household economy as average and 17% as good. The majority (72%) did not answer questions about their partner’s work situation, suggesting they rely on a single income.

Among the mothers who had experienced maltreatment (70%), the median age at which mothers first reported experiencing violence was 20 years (SD = 10.6); for sexual harassment, it was 16 years (SD = 9.5); for rape, it was 18 years (SD = 10.3); and for sexual abuse other than rape, it was 16.5 years (SD = 9.2).

**Table 1:** Overview of participants’ characteristics (*n* = 228)

Variables	*n* (%)
Country of birth	
Norway	175 (77%)
Other	51 (22%)
(Missing)	2
Education	
Primary school, lower secondary school or similar	17 (7%)
Upper secondary education	77 (34%)
Higher education (less than 4 years)	44 (19%)
Higher education (more than 4 years)	41 (18%)
(Missing)	49
Work situation	
Full time work (80–100%)	60 (26%)
Part time work	37 (16%)
Sick leave/disability benefit/work assessment allowance	85 (37%)
Seeking work/in education/Stay-at-home-parent	36 (16%)
(Missing)	10
Yearly income	
Less than 250 000 NOK	21 (9%)
250 000–499 000 NOK	70 (31%)
500 000–749 000 NOK	36 (16%)
750 000–999 000 NOK	15 (7%)
1 000 000 NOK and above	20 (9%)
(Missing)	66
Perceived economic well-being	
Very bad/poor	17 (7%)
Bad/poor	39 (17%)
Average	79 (35%)
Good	38 (17%)
Very good	6 (3%)
(Missing)	49

### Parenting Strategies and Parent-child Relationship Among Non-Offending Mothers

The entire sample of mothers reported a mean score of 14 (SD = 1.6), with 15 being the highest possible score on the positive parenting subscale of the APQ (See Table 2). The mean score in the inconsistent discipline subscale was 7.2 (SD = 3.4) out of a maximum score of 20. For the CPRS subscales, the entire sample achieved a mean score of 30.3 (SD = 4.2) in closeness and 18.5 (SD = 7.0) in conflict, with the highest possible scores of 35 and 40, respectively. The correlation analysis revealed a weak negative correlation (*r*=-0.18) between positive parenting and inconsistent discipline on the APQ and a moderate to strong negative correlation (*r*=-0.52) between closeness and conflict in the CPRS.

The comparison of the two APQ versions, using standardized percentage scores, showed that both subscales were significantly different. For the inconsistent discipline subscale, the reference population from Sollie et al. (2016) study demonstrated a higher standardized mean score (39%) than our sample (36%). The effect size (d = 0.30) was small but statistically significant (*p* = 0.009). For the positive parenting subscale, our sample had a higher standardized mean score (93%) than the reference population (80%). The effect size was large (d=-1.49; *p* < 0.001).

**Table 2:** Mean scores in Alabama Parenting Questionnaire (APQ) and Child Parent Relationship Scale (CPRS) and differences across maternal exposure status

Instrument and subscale	All mothers (*n* = 228)	No maternal exposure (*n* = 68)	Maternal Exposure (*n* = 160)	Statistics	
	Mean (SD)	Mean (SD)	Mean (SD)	*p*-level	Cohen’s d
APQ					
Positive parenting^1^	14.0 (1.6)	13.8 (2.1)	14.1 (1.3)	0.201	0.17
Inconsistent discipline^2^	7.2 (3.4)	7.4 (3.3)	6.6 (3.5)	0.085	0.25
CPRS					
Closeness^3^	30.3 (4.2)	30.5 (4.3)	30.2 (4.1)	0.559	0.08
Conflict^4^	18.5 (7.0)	16.7 (6.3)	19.3 (7.2)	0.009	0.39

^1^ Has 3 items with total possible score of 15; ^2^ Has 4 items with total possible score of 20; ^3^ Has 7 items with total possible score of 35; ^4^ Has 8 items with total possible score of 40

### Differences in Parenting Behaviors Between Mothers with and without Maltreatment Experiences

When comparing mothers with and without maltreatment experiences, there were no significant differences in positive parenting or inconsistent discipline subscale scores (*p* = 0.20 and *p* = 0.085, respectively). Effect sizes also showed no substantial differences between the groups in the APQ subscales. The analysis of the subscales in the CPRS instrument revealed a statistically significant difference between the two groups of mothers on the conflict scale (*p* = 0.009), with mothers who had experienced maltreatment reporting higher levels of conflict in their relationships with their children (See Table 2). The effect size for conflict was small-to-moderate (0.39). The analysis of the closeness subscale revealed no significant difference between the groups (*p* = 0.559).

**Fig. 1:**
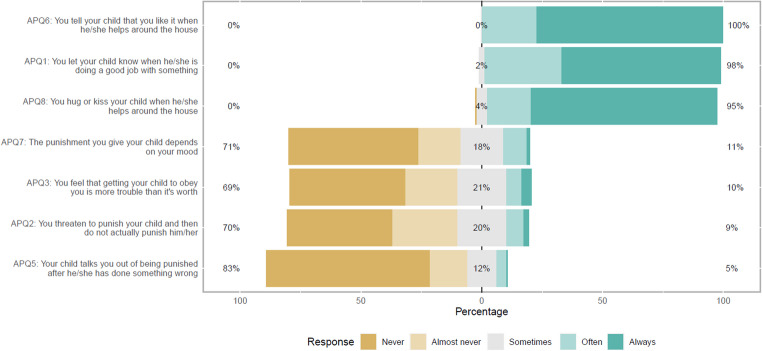
Alabama Parenting Questionnaire (APQ) response distribution for all mothers (entire sample). *Note*: Items APQ1, APQ6 and APQ8 are in the positive parenting subscale. One item (APQ4) was removed from positive parenting. The inconsistent discipline subscale includes items APQ2, APQ3, APQ5 and APQ7

**Fig. 2:**
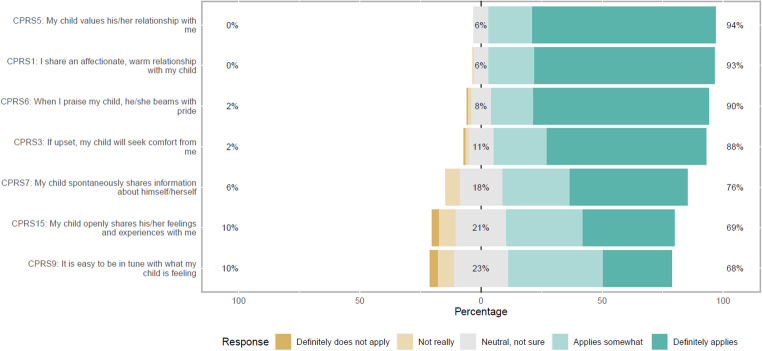
Child-Parent Relationship Scale (CPRS) response distribution for all mothers (entire sample) in the closeness subscale

**Fig. 3:**
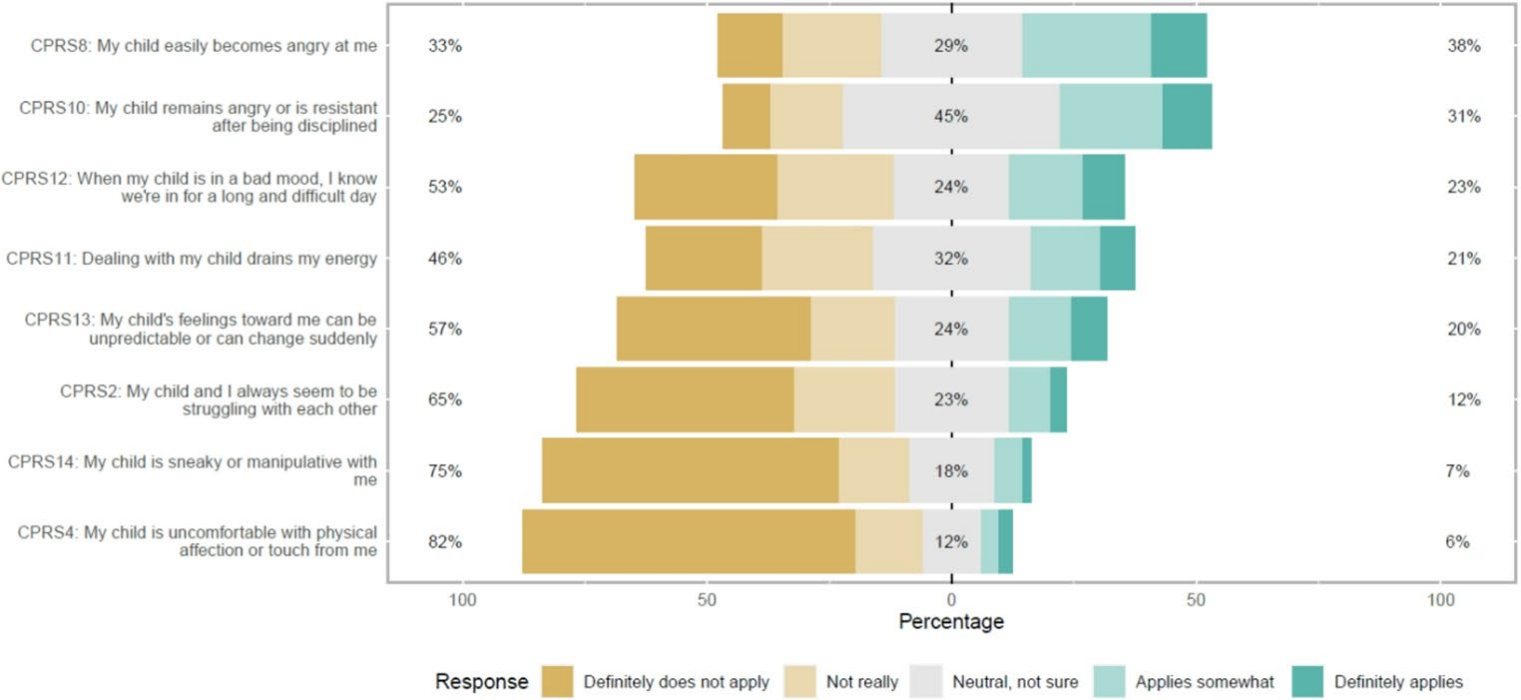
Child-Parent Relationship Scale (CPRS) response distribution for all mothers (entire sample) in the conflict subscale

**Fig. 4:**
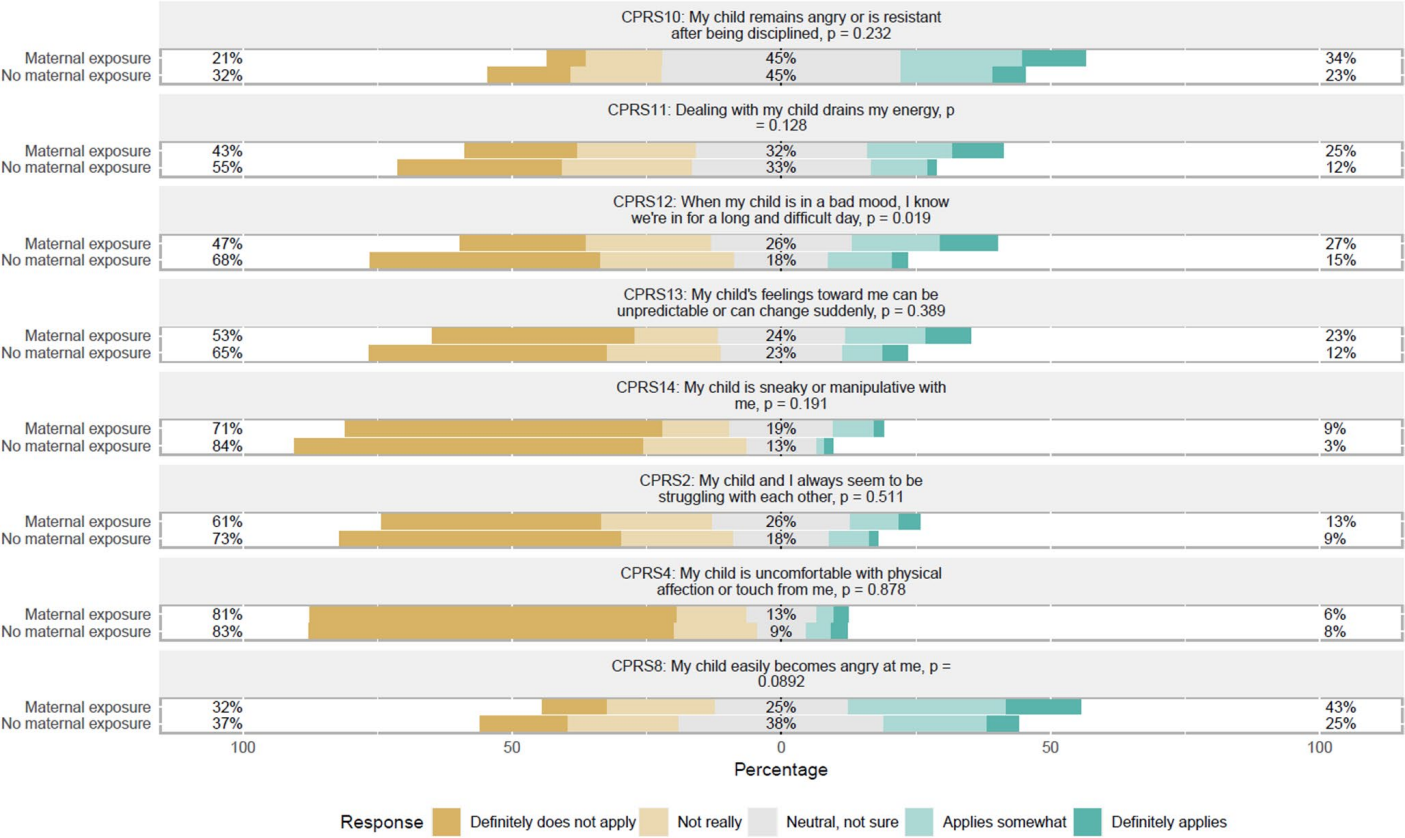
Child-Parent Relationship Scale (CPRS) response distribution in the conflict subscale divided by maternal exposure status

Figures 1, 2, 3 and 4 show the APQ and CPRS response distribution. While Figs. 1, 2 and 3 show the response distribution for the entire sample, Fig. 4 compares the response distribution of the two groups of mothers. Figure 1 shows the response for all items in both subscales of the APQ, while Figs. 2 and 3 show the response distribution for all items in the closeness and conflict subscales, respectively, from CPRS. Figure 4 shows the response distribution for conflict, the only subscale showing significant differences between the groups.

As shown in Fig. 1, most mothers responded “always” or “often” to the positive parenting statements. For instance, in response to the statement “You tell your child that you like it when he/she helps around the house”, 100% responded “always” or “often”. Similarly, 98% answered that they always or often let their child know when they are doing a good job with something. The responses to the statements in inconsistent discipline were mostly “never or “almost never”. For example, to the statement “Your child talks you out of being punished after he/she has done something wrong”, 83% responded “never” or “almost never”. The majority (71%) also reported that their punishments never or almost never depend on their mood.

Figure 2 shows a similar pattern in the CRPS closeness subscale, with the mothers responding similarly to the statements. For example, 94% reported that it “definitely applies” or “applies somewhat” that their child values their relationship with them. None reported that it did not apply, which was the case with the statement “I share an affectionate, warm relationship with my child”. While 88% responded that it definitely applies or somewhat applies that their child seeks comfort from them if upset, only 2% reported that this did not apply. Some statements illustrated more variation in the responses, such as the statement “It is easy to be in tune with what my child is feeling”, with 68% reporting “applies somewhat” or “definitely applies”. In comparison, 23% were neutral or not sure.

The illustration of the conflict subscale of the CPRS showed more variation in the mothers’ responses (see Fig. 3). For instance, to the statement “My child remains angry or is resistant after being disciplined”, 45% responded “neutral, not sure”. In comparison, 25% responded that it definitely or not really applied, and 31% responded that it definitely or somewhat applied. As shown in Fig. 4, most items in the conflict subscale did not show a statistically significant difference between the groups. However, the statement, “When my child is in a bad mood, I know we’re in for a long and difficult day,” showed a statistically significant difference (*p* = 0.019), with 27% of the Maternal exposure group agreeing, compared to 12% of the No maternal exposure group. For other statements, such as “My child and I always seem to be struggling with each other” and “My child is uncomfortable with physical affection or touch from me”, the groups responded similarly, with no significant differences (*p* > 0.05).

## Discussion

In this study, we examined characteristics of parenting strategies and the parent-child relationship among non-offending mothers of children who have been exposed to violence and abuse. We also investigated whether there were differences in parenting behaviors between mothers with and without maltreatment histories.

The participants in our study reported high mean scores in positive parenting and closeness. High scores in these subscales indicate a parenting practice characterized by positive involvement with their child and that they perceive their relationship with their child to be characterized by warmth, love, and open communication. The analysis of item-level responses revealed that the mothers use positive reinforcement behaviors and have strong and affectionate relationships with their children. For instance, 100% reported that they always or often tell their child that they like it when they help around the house. The mothers also showed low mean scores in inconsistent discipline, suggesting they have a consistent approach to discipline and follow through with consequences for misbehavior. For example, 83% responded that they never or almost never let their child talk them out of punishment. On the other hand, the mothers achieved relatively high scores in conflict, which indicates that they perceive their relationship with their child to be somewhat characterized by negativity.

Our findings contrast with studies from other countries where participants reported less affectionate parenting styles, lower levels of care, more negative parenting, and poorer parent-child relationships in families where a child has experienced maltreatment (Nilsson et al., 2017; Pelcovitz et al., 2000; Sunday et al., 2008; Cabbigat & Kangas, 2018). These differences may be attributed to variation in sample characteristics, such as child age, whether samples included mothers only or both mothers and fathers, access to welfare services, socioeconomic backgrounds and cultural factors. For example, a study examining parents’ perceptions of their relationships with their children during the transition from preschool to elementary school found that both mothers and fathers reported higher levels of closeness and lower levels of conflict when their children were preschool-aged compared to the elementary school period (Driscoll & Pianta, 2011). In addition, mothers consistently reported higher levels of both closeness and conflict than fathers. This pattern may help explain differences between our findings and those from other studies that include fathers and children in different age groups. Moreover, parent and child experiences and perspectives on parenting and family relations may differ. While we have used self-reported information from mothers, other studies such as Nilsson et al. (2017) used self-reported information from adolescents.

Comparing APQ scores across studies presents challenges due to a lack of established normative data (Essau et al., 2006). To address this, we standardized mean scores for the APQ subscales based on their maximum possible scores, enabling comparison with results from Sollie et al. (2016). This way, we could compare the results despite differences in item counts. Mothers in our study had a mean score of 14, out of 15, in positive parenting and 7.2, out of 30, in inconsistent discipline. In contrast, the Norwegian validation study of the 42-item APQ (Sollie et al., 2016), the participating mothers from the reference population (*N* = 78) had a mean positive parenting score of 24.81 (SD = 3.05) out of a possible 30. The mean inconsistent discipline score was 14.47 (SD = 3.46) out of 30. The standardized means and effect sizes in both subscales revealed significant differences between our and the reference population. For inconsistent discipline, the reference population demonstrated a higher standardized mean score (39%) than ours (36%). The difference was modest but statistically significant. Our sample had a higher standardized mean score (93%) for positive parenting than the reference population (80%), indicating a substantial difference. Still, these results may also reflect differences in construct representation due to item count variations and differences in factor loadings across items in the different versions. Potential congeneric model effects should be borne in mind when considering the presented results (Czerwiński & Atroszko, 2023).

The CPRS scores reported by our participants align with those of a control group in another Norwegian study including parents of children aged 0–18 years (M = 5.2 years). The control group reported a baseline mean score of 29.3 (SD = 4.1) for closeness and 18.9 (SD = 5.2) for conflict (Brekke et al., 2023). The mothers in our study achieved a slightly higher closeness mean score (30.3) and a similar mean score in conflict (18.5). However, it is worth noting that the control group from Brekke et al. (2023) included parents who had signed up and were on the waitlist to receive the universal version of the International Child Development Programme (ICDP). The ICDP’s target group is all parents, and it aims to empower them to master parenthood (ICDP Norge, 2024). The control group could, therefore, include parents struggling in the parental role. Furthermore, 22.5% of the participating parents were fathers (Brekke et al., 2023), which may have influenced the overall scores due to sex differences in parenting styles. For example, a systematic review of 31 studies from more than 15 countries found that mothers were more accepting, responsive, and supportive than fathers (Yaffe, 2023). A recent study on parenting practices in Norway found that although the participants generally reported high levels of warmth and support, mothers reported slightly higher levels of these constructs, while fathers reported slightly higher incidences of rejection, chaos, and coercion (Nygaard et al., 2024). These differences between mothers and fathers may explain why our sample, consisting solely of mothers, reported higher closeness scores than the control group from the Brekke et al. (2023) study.

Our comparison of the Maternal exposure and No maternal exposure groups only revealed a difference in conflict, where mothers with violence and abuse experiences reported experiencing more conflict in their relationship with their children. When comparing response distribution in the conflict subscale, we found a significant difference between the groups regarding their experiences with their child’s moods. A larger proportion of the Maternal exposure group anticipated that days when their child is in a bad mood would often feel long and challenging. There were no significant differences between the groups in the other domains (positive parenting, closeness, and inconsistent discipline), with both groups reporting having a close emotional bond with their child, using positive parenting strategies, and having a consistent approach to discipline.

Other studies investigating the parenting of mothers with maltreatment experiences have found that this group of mothers may experience parenting difficulties and have difficulty being nurturing and available (Greene et al., 2020), which contradicts our findings. However, the mothers participating in our study, particularly the mothers with maltreatment experiences, did report relatively high levels of conflict, which is similar to the findings of some studies included in the review from Greene et al. (2020). One study found that mothers with a history of child sexual trauma (CST) were more likely to exhibit harsh and intrusive parenting behaviors than mothers without a history of CST (Zvara et al., 2017). Other studies concluded that a history of child sexual abuse (CSA) may lead to emotional distance in the parent-child relationship and that interpersonal traumatic experienced by mothers increased the risk of verbal hostility and physical coercion (Ehrensaft et al., 2015; Schwerdtfeger et al., 2013). In a study including non-offending parents, participants reported experiencing a range of emotions in response to their children’s CSA experiences, such as guilt and regret, betrayal and helplessness, and depression and anxiety (Vilvens et al., 2021). This study also found that lack of social support or untreated mental health issues could decrease the parents’ capacity to successfully cope with their child’s abuse, which can diminish their ability to stay emotionally and cognitively connected with their child. Our findings align with prior research on differences between mothers and fathers, which suggests that occurrences of both negative and positive emotional expressions and interactions are more frequent in mothers-/child relationships than in fathers-child relationships (Yan et al., 2019; Driscoll & Pianta, 2011). Mothers tend to be more accepting, responsive, and supportive than fathers, but may also be more controlling and demanding (Yaffe, 2023). As our sample includes only mothers, this pattern may partly explain the coexistence of positive parenting, closeness, consistent discipline, and conflict.

Our findings expand on the current literature on parenting styles and practices which have mainly been conducted in Anglo-American countries (Nygaard et al., 2024). The Nordic countries differ from many other societies, notably through extensive welfare services and progressive parenting politics promoting equality in parenthood (Grødem, 2015). Norwegian parents are also typically focused on collaboration and engaging in dialogue with their children and avoid using coercive measures (Hollekim et al., 2016). This may also explain some of the differences between our findings and other studies. The previously mentioned study on parenting practices in Norway also had findings that contradicted other studies; parents having lower incomes reported more supportive emotion-socializing practices than those with higher incomes (Nygaard et al., 2024). As pointed out by Nygaard et al., this is the opposite of what some previous empirical studies from other countries have found (Berger et al., 2009; Ayoub & Bachir, 2025).

When interpreting the findings, it is important to consider the representativeness of the sample in comparison to the general population. Compared to the general population of Norwegian women in the same age group, a larger percentage of our participants had upper secondary education as their highest level of education attained, and fewer had higher education (Statistics Norway, 2024a). They also reported working less fulltime and part-time than the general population, with a larger percentage reporting being on sick leave, disability benefits, or work assessment allowance (Statistics Norway, 2024b). The majority reported having a yearly income between 250,000 and 499,000 Norwegian kroner (31%; *n* = 70). In 2023, the average yearly salary of women in Norway was 630 360 Norwegian kroner (Statistics Norway, 2024b), or roughly 54,000 Euros. Still, the majority (52%) of the mothers reported that they perceived their economic well-being as good or average. Overall, the mothers had lower socio-economic status than the general female population in the same age group. This may be a result from increased caregiving demands, which can influence socioeconomic status (Freeman & Dodson, 2021; Bamber et al., 2023). It might also be a consequence from having experienced maltreatment themselves (Herbert et al., 2023).

Lastly, the findings in this study are encouraging. The mothers participating in this study reported high levels of closeness with their children and having positive parenting strategies and consistent discipline techniques. Considering that a stable and secure relationship between the child and their caregiver can moderate the impact of their maltreatment experiences (Harden et al., 2024), these findings are particularly promising.

### Strengths and Limitations

We have managed to examine the characteristics of a group that is often difficult to identify and recruit in research, which is a strength of the current study. Studies on childhood maltreatment frequently face significant obstacles, including the risk of emotional strain for participants, legal and ethical restrictions, the requirement for parental consent involving minors, and the societal taboos surrounding the topic (Chae et al., 2011; Kinard, 2001). Our sample includes mothers of children with a history of maltreatment, substantiated by either a primary physician, Child Welfare Services, or other officials. The mothers have also chosen to apply for participation in the one-week course at the SSC. This introduces a limitation to the generalizability of our findings in that the results may not extend to mothers who have not sought help or engaged with support services or to families where maltreatment has not been formally identified. There are some additional limitations that must be acknowledged in this study. Several items in the positive parenting subscale of the APQ show little variability. Similar patterns of limited variability have been reported in other Norwegian studies with comparable sample sizes (Sollie et al., 2016; Brekke et al., 2023), suggesting that this response pattern is not unique to the present study. However, the degree of variability observed here seems to be somewhat lower, which may at least partly be related to the use of a shortened version of the instrument and the removal of one item due to low reliability. Low variability has also been reported in international studies, such as Gross et al. (2017), indicating that this pattern is not specific to the Norwegian version of the scale. Furthermore, although sample size and statistical power are unlikely to fully explain the observed pattern, they may still represent contributing factors. Taken together, it is difficult to determine the extent to which the low variability reflects sample characteristics, measurement properties, or contextual factors, and these issues should be taken into account when interpreting the findings.

Comparing our findings to those of other studies is challenging because research on child maltreatment and parenting practices is scarce. There are also variations in definitions, methods, and sample characteristics in the available studies, which further hampers an overview of the research field (van IJzendoorn et al., 2020; Jackson, 2023). These inconsistencies not only complicate the interpretation of individual findings but also comparisons across studies. As highlighted in our discussion, factors such as the inclusion of both genders versus only mothers and the use of self-reported data from parents versus children or adolescents add further complexity to these comparisons. Furthermore, the directionality of the associations between child maltreatment and parenting is uncertain. Risk factors within families may simultaneously increase the likelihood of negative parenting practices and child maltreatment. Future longitudinal studies are needed to entangle the temporal order of these factors and to better understand how they interact and influence each other over time. Moreover, parenting practices are likely influenced by additional factors beyond those explored in this study, such as socioeconomic background, parental mental health, culture, and social support (Roubinov & Boyce, 2017; Fung et al., 2018; Sanders & Turner, 2018).

### Implications

Our findings have important implications for clinical practice and the development of targeted interventions for families affected by maltreatment. The fact that mothers in this study demonstrated positive parenting practices and strong emotional bonds with their children despite adverse circumstances underscores their resilience. Clinicians could build upon these strengths to foster further positive parenting and enhance the parent-child relationship. The elevated conflict level experienced among mothers with experiences of violence and abuse highlights a potential area of intervention. For example, one approach to support these mothers could be tailored parenting programs that focus on managing parent-child conflict in families affected by maltreatment. By addressing these challenges while leveraging the strengths demonstrated by the mothers in this study, clinicians can contribute to creating more secure and nurturing environments for children, potentially reducing the intergenerational transmission of trauma and improving long-term outcomes for these families.

## Conclusion

In this study, we have explored parenting strategies and the parent-child relationship among non-offending mothers of children who have been exposed to maltreatment. Our findings suggest that, despite the children’s experiences and the challenges that might follow, the mothers view their relationship with their children as warm and affectionate. They also reported that they communicate well with their children, use positive reinforcement strategies and are consistent in their discipline techniques. However, mothers who themselves had been exposed to violence and abuse reported higher levels of conflict in the parent-child relationship, emphasizing the potential challenges these mothers may face in managing the emotional and behavioral dynamics with their children. This aligns with previous research suggesting that parental trauma can influence parenting practices. Summarized, our findings underscore the resilience and positive practices of the mothers while highlighting areas of potential challenge, offering critical insights for designing targeted interventions and informing policies aimed at supporting families affected by maltreatment.

Our findings also underscore the importance of cultural and contextual factors when interpreting parenting practices. Parenting practices in the Nordic context appear to diverge from those observed in other cultural settings. This highlights the need for more research to better understand how societal factors shape parenting and gain a nuanced understanding of parenting among mothers of children exposed to violence and abuse.

## Data Availability

Norwegian data protection regulations and GDPR impose restrictions on sharing of individual participant data. Researchers may gain access to survey participant data by contacting the publication committee (borge.sivertsen@fhi.no). Approval from the Norwegian Regional Committee for Medical and Health Research Ethics (REK) (https://rekportalen.no) is a pre-requirement to access the data. The Norwegian Institute of Public Health (NIPH) manages the dataset and guidelines for access are available at https://www.fhi.no/en/hd/access-to-data/.
